# From the plate to the brain: associations between dietary patterns and reduced dementia prevalence and white matter lesions in older Japanese adults

**DOI:** 10.1007/s11357-025-01791-7

**Published:** 2025-07-29

**Authors:** Liying Chen, Yasuko Tatewaki, Benjamin Thyreau, Kazuhiro Uchida, Hikari Iki, Shigeyuki Nakaji, Tetsuya Maeda, Kenjiro Ono, Moeko Noguchi-Shinohara, Masaru Mimura, Kenji Nakashima, Jun-ichi Iga, Minoru Takebayashi, Toshiharu Ninomiya, Yasuyuki Taki, Japan Prospective Studies Collaboration for Aging, Dementia (JPSC-AD) study group, Jun Hata, Mao Shibata, Takanori Honda, Tomoyuki Ohara, Masato Akiyama, Koichi Murashita, Tatsuya Mikami, Songee Jung, Mina Misawa, Naoki Ishizuka, Hiroshi Akasaka, Yasuo Terayama, Hisashi Yonezawa, Junko Takahashi, Masahito Yamada, Kazuo Iwasa, Sohshi Yuki-Nozaki, Shogyoku Bun, Hidehito Niimura, Ryo Shikimoto, Hisashi Kida, Yasuyo Fukada, Hisanori Kowa, Kenji Wada, Masafumi Kishi, Takaaki Mori, Yuta Yoshino, Hideaki Shimizu, Ayumi Tachibana, Shu-ichi Ueno, Tomohisa Ishikawa, Ryuji Fukuhara, Asuka Koyama, Mamoru Hashimoto, Manabu Ikeda, Yoshihiro Kokubo, Midori Esaki, Yuji Takano, Koji Yonemoto, Hisako Yoshida, Kaori Muto, Yusuke Inoue, Yukihide Momozawa, Chikashi Terao, Michiaki Kubo, Yutaka Kiyohara

**Affiliations:** 1 Department of Aging Research and Geriatric Medicine, Institute of Development, Aging and Cancer, Tohoku University, Sendai, Japan; 2 Smart-Aging Research Center, Tohoku University, Sendai, Japan; 3 Department of Health Promotion, School of Health and Nutrition Sciences, Nakamura Gakuen University, Fukuoka, Japan; 4 Department of Social Medicine, GraduateSchool of Medicine, Hirosaki University, Hirosaki, Japan; 5 Division of Neurology and Gerontology, Department of Internal Medicine, School of Medicine, Iwate Medical University, Yahaba, Japan; 6 Department of Neurology, Kanazawa University Graduate School of Medical Sciences, Kanazawa, Japan; 7 Department of Neuropsychiatry, Keio University School of Medicine, Tokyo, Japan; 8 National Hospital Organization, Matsue Medical Center, Shimane, Japan; 9 Department of Neuropsychiatry, Molecules and Function, Ehime University Graduate School of Medicine, Shitsukawa, Ehime, Japan; 10 Department of Psychiatry and Neuroscience, Center for Metabolic Regulation of Healthy Aging, Faculty of Life Sciences, Kumamoto University, Kumamoto, Japan; 11 Department of Epidemiology and Public Health, Graduate School of Medical Sciences, Kyushu University, Fukuoka, Japan; 12 Japan Prospective Studies Collaboration for Aging and Dementia (JPSC-AD), Fukuoka, Japan

**Keywords:** Dietary pattern, Dementia, White matter lesions, Older people

## Abstract

Diet is widely considered essential in dementia, but its association with white matter lesions (WMLs) remains unclear. This cross-sectional study investigated the associations between dietary patterns, dementia, and WMLs in a large, nationwide, multicenter population of older Japanese adults. A total of 8,938 adults (aged ≥ 65; 73 ± 6.3 years old) from the Japan Prospective Studies Collaboration for Aging and Dementia (JPSC-AD) were included. Dietary intake was assessed using a Food Frequency Questionnaire. Principal component analysis was used to derive dietary patterns. A trained Convolutional Neural Network model segmented WMLs from brain MR images. Logistic regression estimated odds ratios (ORs) for dementia by dietary pattern quartiles, while linear regression assessed associations with WML volumes. Five dietary patterns were extracted. A Japanese diet including protein and minerals was significantly associated with lower prevalence of all-cause dementia (OR = 0.56) and Alzheimer’s disease (OR = 0.47), and with reduced WML volume (*β* = − 0.03). Similar directional trends in ORs were observed across study sites. The reverse association with WMLs remained significant among individuals without dementia, reducing the likelihood of reverse causation. A Japanese diet including protein and minerals was associated with lower dementia prevalence and smaller WML volume in older Japanese adults. Drawing on nationwide, large-scale, multicenter data, these findings advance our understanding of dietary patterns in older Japanese adults and provide valuable insights for future intervention studies targeting diet and age-related brain changes.

**Supplementary Information:**

The online version contains supplementary material available at 10.1007/s11357-025-01791-7.

## Introduction

Dementia affects approximately 50 million people worldwide, with 10 million new cases reported annually [1]. As super-aged societies increase, it has become an urgent public health concern [2, 3]. Consequently, developing effective treatment and prevention strategies for dementia is critical to maintaining the patients’ quality of life and softening societal burdens.

White matter lesions (WMLs) are common in late life and appear as hyperintensities on T2-weighted magnetic resonance (MR) images, often resulting from ischemia, inflammation, degenerative changes, infections, etc. [4, 5]. Beyond their diagnostic relevance, WMLs are now recognized as structural imaging markers of brain aging. Quantitative analysis of WMLs helps assess disease progression and severity [6], and they are considered a key indicator of small vessel disease as well as a known risk factor for cognitive decline and dementia.

Compared to other lifestyle factors, diet is more accessible to modify for older people. The relationship between dietary patterns and dementia has gained increasing attention. Three dietary patterns have been associated with dementia prevention. The Mediterranean Diet (MeDi), rich in unsaturated fatty acids, PUFAs (Polyunsaturated Fatty Acids), antioxidants, vitamins, and minerals [7, 8]; the Dietary Approaches to Stop Hypertension (DASH), low in saturated fat and sugar [9]; and the Mediterranean-DASH Intervention for Neurodegenerative Delay (MIND), combines MeDi and DASH diets [10]. However, these diets are largely derived from Northern European dietary practices and may not align well with traditional East Asian food culture [11], potentially making long-term adherence difficult and limiting potential cognitive benefits in East Asian populations. The dietary habits and ethnic differences make it necessary to study the impact of dietary patterns on dementia within East Asian cultural contexts.

In this context, Japan has the fastest-aging population in the world, with over 30% projected to be ≥ 65 by 2025 [12], highlighting the urgent need to address aging-related health issues such as dementia. Although several studies have explored the association between dietary patterns and dementia in Japan, including the Hisayama Study, which reported that a dietary pattern rich in soy products, vegetables, algae, and dairy, and low in rice, was associated with reduced dementia risk [13]. However, most previous studies have been limited to single-region samples, and none have examined these associations using a standardized Food Frequency Questionnaire (FFQ) across a nationwide, multicenter population. Furthermore, few have investigated the relationship between dietary patterns and dementia using brain imaging markers.

Given that dietary behavior is deeply shaped by cultural and regional factors, it is essential to investigate whether dietary patterns are associated with brain health in a culturally distinct sample. Although prior studies have shown associations between diet and cognitive health, these findings may not be directly applicable to the Japanese population due to cultural dietary differences. There is a clear need for large-scale, multicenter studies using standardized FFQ and high-resolution brain imaging to clarify the relationship between culturally embedded dietary structures and markers of brain integrity among older adults in Japan.

This study examined the relationship between dietary patterns, dementia, and WMLs in a multicenter sample of 11,957 older Japanese adults (8,938 included in the final analysis after exclusion criteria were applied). It is notable for incorporating quantitative WMLs as structural markers of brain aging and implementing a standardized nationwide protocol, which enables comparisons across study sites and offers a more comprehensive understanding of these associations across Japan.

## Materials and methods

### Study population and exclusion criteria

This study utilized data from the Japan Prospective Studies Collaboration for Aging and Dementia (JSPC-AD), which collected data from 11,957 participants across eight sites in Japan: Nakayama (Ehime), Hirosaki (Aomori), Hisayama (Fukuoka), Yahaba (Iwate), Nakajima (Ishikawa), Arakawa (Tokyo), Arao (Kumamoto), and Ama (Shimane). The baseline survey for the JPSC-AD was conducted from 2016 to 2018. A detailed description of the baseline survey was published elsewhere [14].

Participants were excluded based on the following criteria: (1) failure to pass FreeSurfer quality control, (2) extreme outliers in energy intake, (3) age < 65 years, and (4) missing data for key covariates (education, diabetes, hypertension, blood sample, history of stroke, BMI, smoking habits, and exercise habits). These exclusion criteria ensure the reliability of the study population.

### Diagnosis of dementia

Dementia was diagnosed according to the criteria of the Diagnostic and Statistical Manual of Mental Disorders, Revised Third Edition (DSM-III-R). Alzheimer’s disease (AD) was identified based on the criteria established by the National Institute of Neurological and Communicative Disorders and Stroke and the Alzheimer’s Disease and Related Disorders Association (NINCDS-ADRDA), while vascular dementia (VaD) was diagnosed using the National Institute of Neurological Disorders and Stroke–Association Internationale pour la Recherche et l’Enseignement en Neurosciences (NINDS-AIREN) criteria [14].

The diagnosis of dementia was established through a standardized two-step diagnostic protocol, uniformly applied across eight research institutes to ensure diagnostic consistency. In the first step, cognitive screening was conducted using the Mini-Mental State Examination (MMSE). Participants meeting any of the following criteria were selected for further evaluation: (1) MMSE score ≤ 26, (2) delayed recall score ≤ 4 out of 6, (3) failure in the intersecting pentagon-copying or cube-copying tests, and (4) suspected cognitive impairment based on verbal and behavioral performance.

In the second step, expert psychiatrists or neurologists conducted comprehensive physical and neurological examinations to determine the presence of mild cognitive impairment (MCI) or dementia, as well as to classify dementia subtypes. These evaluations incorporated the delayed recall test from the Logical Memory IIA subscale of the Wechsler Memory Scale-Revised and the Pareidolia test. Supplementary information was gathered through patient interviews, family members, primary care physicians, medical records, and brain imaging results. Cutoff scores for cognitive impairment in the Wechsler Memory Scale-Revised were adjusted according to participants’ education levels: ≤ 8 points (≥ 16 years of education), ≤ 4 points (8–15 years), and ≤ 2 points (≤ 7 years).

To maintain diagnostic consistency across participating cohorts, all assessments followed the same standardized criteria, and dementia diagnoses were confirmed through multidisciplinary expert evaluations at each research institute.

Among the participants included in the analysis, 359 were identified as having all-cause dementia. Of these, 292 were diagnosed with AD and 64 with VaD.

### Dietary assessment and food grouping

Dietary intake was conducted using an FFQ, which consists of 215 questionnaire items that survey the frequency of eating and drinking per week and the amount per serving (little, normal, much) for 65 foods and beverages classified from a list of 233 items (Available at: https://www.eph.med.kyushu-u.ac.jp/jpsc/link/pdf/ffq01.pdf).

The daily intake of each food and nutrient was calculated based on FFQ responses, and nutrient values were calculated based on the Japanese Standard Tables of Food Composition 2015, 7th Revised Edition. The validity of the FFQ was evaluated by comparing FFQ-derived estimates with values obtained from 4-day weighed dietary records conducted across all four seasons. Pearson’s correlation coefficients were used to assess agreement between the two methods (Available at: https://www.eph.med.kyushu-u.ac.jp/jpsc/link/pdf/ffq02.pdf).

To reduce data complexity, foods were grouped according to similarities in nutrient content and culinary use, based on the nutrient profiles listed in the Food Composition Tables of Japanese Foods, 5th Revised Edition. In addition, food groupings were aligned with the categories used in the National Nutrition Survey in Japan to ensure consistency with commonly used nutrient profiles and cooking practices. Consequently, 18 food groups were established (Supplementary data 1) and used in subsequent analyses of dietary patterns.

### Brain MRI analysis

**Fig. 1:**
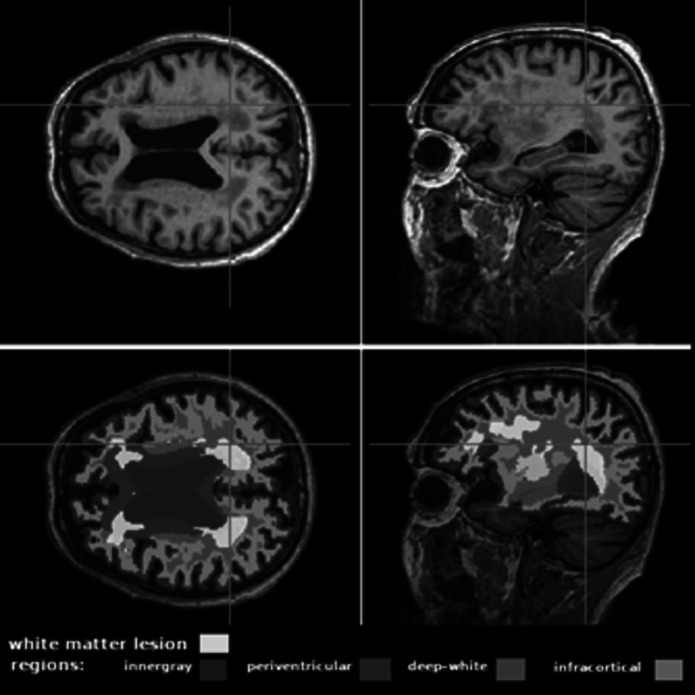
Perform WML segmentation using the developed ConvNet. The illustration shows axial and sagittal views of a T1-weighted MRI brain scan of a 76-year-old female participant. The top two images show the original MRI scan without segmentation, and the bottom two images show the automatic segmentation results from ConvNet Regions of interest (ROIs) are marked in blue, and WMLs are highlighted in yellow

T1-weighted 3D images were acquired according to the brain MRI protocol of the Alzheimer’s Disease Neuroimaging Initiative study [15]. A trained 3D-T1 Convolutional Neural Network (ConvNet) was used to obtain the quantitative volume of WMLs. Briefly, the ConvNet had been previously trained to extract WML masks directly from T1-weighted images, rather than FLAIR sequence. The trained ConvNet model is available at https://github.com/bthyreau/deep-T1-WMH. Further information on the development and validation of this ConvNet model is available in previously published studies. [16]. In addition, considering that lesion etiology may vary across brain regions, WMLs were classified based on anatomically defined regions of interest: periventricular (PWML), deep (DWML), and infracortical (IWML) (Fig. 1).

### Assessment of lifestyle variables and confounding factors

At baseline, participants completed a questionnaire on medical history, diabetes and hypertension treatment, education, smoking, and physical activity habits. Stroke history was defined as a preexisting sudden onset of nonconvulsive and persisting focal neurologic deficit. Education was classified into four levels: low (< 6 years), low-intermediate (7–9 years), intermediate (10–12 years), and high (> 13 years). Smoking status was categorized as never, former, or current. Exercise was defined as activity ≥ 30 min/session, ≥ 2 times/week, for ≥ 1 year. Hypertension was defined as blood pressure ≥ 140/90 mm Hg or antihypertensive medication use. Body mass index (BMI, kg/m^2^) was calculated from height and weight measured in light clothing without shoes. Diabetes was diagnosed based on ADA 2010 criteria [17].

### Statistical analysis

Principal component analysis (PCA) was used to determine the food pattern based on the 18 food groups. Food intake (g/d) was adjusted using a density method and log-transformed to correct skewness. PCA suitability was confirmed by Kaiser–Meyer–Olkin and Bartlett’s sphericity tests. Components with eigenvalues > 1.0 were retained as the most widely used. After Varimax rotation, factor scores were saved from the PCA for each individual. The scores were divided into quartiles to facilitate subsequent comparisons of dietary patterns. Food groups with factor loadings > 0.2 were used to characterize each dietary pattern as common practice [18].

In the descriptive analysis, generalized linear models were used to analyze continuous variables, and chi-square tests were applied to categorical variables. Logistic regression analysis was performed to calculate the odds ratio (ORs) and 95% confidence interval (95% CI) of dementia for each quartile of the dietary pattern. Multivariate adjusted ORs were calculated by adjusting for age, sex, education, diabetes, hypertension, total cholesterol, history of stroke, BMI, smoking habits, exercise habits, and energy intake.

Additionally, site-specific analyses were performed for each of the eight sites. This allowed for the assessment of variations in dietary habits across areas and improved the interpretability of the results.

Log-transformed volumes of WMLs were analyzed using multiple linear regression to examine associations between dietary patterns and WMLs, with the regression coefficient (β) representing the effect of a one-quartile increase in dietary pattern scores. Two models were constructed. Model 1 included the estimated total intracranial volume (eTIV), age, sex, and study site. Model 2 was further adjusted for education, diabetes, hypertension, total cholesterol, history of stroke, BMI, smoking habits, exercise habits, and energy intake.

Moreover, we conducted a sensitivity analysis in a subset of individuals without dementia. This approach aimed to evaluate whether the observed associations between dietary patterns and WMLs remained consistent in this group, thereby minimizing the potential impact of reverse causation. This consideration is particularly important given the cross-sectional design of the study and the possibility that dietary changes may occur during the prodromal phase of dementia.

All statistical analyses were conducted using SPSS version 28 (IBM, Armonk, NY, USA). A two-sided *P*-value = 0.05 was considered significant. Plots were generated using R version 4.5.0 (R Foundation for Statistical Computing, Vienna, Austria).

## Results

### Participant selection

**Fig. 2:**
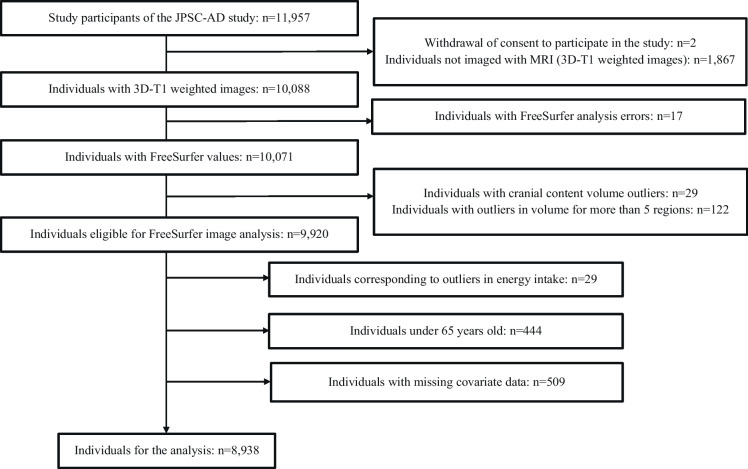
Participant selection flowchart for the JPSC-AD study. The flowchart shows the inclusion of 8,938 participants from an initial 11,957 based on MRI data quality, age, energy intake, and covariate completeness

Among the 11,957 participants enrolled in the JPSC-AD, 8,938 individuals (73 ± 6.3 years) were included in the final analysis after applying exclusion criteria based on MRI quality, energy intake outliers, age, and missing covariates (Fig. 2). Among these, 42.7% were male, and 4.0% were diagnosed with dementia.

### Dietary pattern analysis

Overall, five dietary patterns accounted for 45.7% of the variance in food intake. The factor loading matrices are shown in Table 1. Factor loadings indicate the magnitude and direction of each food group’s contribution to the dietary pattern score. Positive factor loadings indicate that the higher the dietary pattern score, the higher the intake of that food group.

**Table 1:** Principal components and corresponding scoring coefficients for dietary variables (Principal component analysis was used to determine dietary patterns)

Food groups	Principal components				
	Principal component 1 (Dietary pattern 1)	Principal component 2 (Dietary pattern 2)	Principal component 3 (Dietary pattern 3)	Principal component 4 (Dietary pattern 4)	Principal component 5 (Dietary pattern 5)
Green vegetables	0.72	−0.06	0.10	0.12	−0.17
Other vegetables	0.71	−0.14	0.09	0.15	−0.07
Algae	0.63	0.00	0.04	−0.04	−0.03
Potatoes	0.52	−0.04	0.17	0.14	0.15
Fish	0.49	0.09	−0.28	−0.03	0.04
Rice	−0.06	−0.75	0.08	−0.16	0.17
Noodles and other cereals	−0.05	0.68	−0.12	−0.16	0.23
Bread	−0.15	0.58	0.32	0.27	−0.18
Alcoholic drinks	−0.23	0.16	−0.65	−0.13	0.01
Sugar and confectioneries	−0.21	−0.06	0.59	0.03	0.33
Fruits and fruit juices	0.35	0.20	0.49	−0.15	−0.11
Fats and oils	−0.06	0.23	0.17	0.70	0.06
Egg	0.23	−0.14	0.01	0.55	0.00
Meat	0.25	0.16	−0.23	0.38	−0.01
Pickles	0.27	0.11	0.08	−0.17	0.62
Soybeans and soybean products	0.22	0.06	−0.06	−0.17	−0.50
Milk and dairy products	0.22	0.22	0.39	−0.09	−0.50
Miso	0.19	−0.25	0.11	−0.23	0.26
Variance explained, %	14.88	10.53	7.95	6.36	6.01

### Features of DP1

For dietary pattern 1 (DP1), food groups with factor loadings of 0.2 included green and other vegetables, algae, potatoes, fish, fruits and fruit juices, egg, meat, pickles, soybeans and soybean products, milk and dairy products, whereas foods with a factor loading < − 0.2 included alcoholic drinks, sugar and confectioneries.

An absolute value threshold of 0.2 indicates strong associations with DP1, but does not exclude the presence of other food items with weaker associations. For example, rice was also part of this dietary pattern, though it showed a negative factor loading. Given that modern Japanese diets have been shifting towards increased consumption of animal-based proteins and dairy products, DP1 was also considered a “Japanese diet including protein and minerals.”

### Population characteristics of DP1

Subjects were divided into quartiles according to the factor score of each dietary pattern. The clinical characteristics of the study population, stratified by score quartiles for the DP1, are presented in Table 2. Compared to the lowest (Quartile 1), participants in the highest (Quartile 4) of DP1 scores were more likely to be female, older, exercise regularly, have higher energy intake, and be less likely to smoke. No significant associations were observed with hypertension or diabetes.

**Table 2:** Clinical characteristics of the study population according to the score quartiles for the dietary pattern 1

Variables	Score for the dietary pattern 1				
	Quartile 1	Quartile 2	Quartile 3	Quartile 4	*p*-value
	(n = 2235)	(n = 2234)	(n = 2235)	(n = 2234)	
	< − 0.55	− 0.55 to 0.13	0.13 to 0.69	≥ 0.69	
Median value of score for the dietary pattern 1	− 1.1	− 0.2	0.4	1.0	< 0.05
Age, years	72 ± 6.0	73 ± 6.2	74 ± 6.3	74 ± 6.3	< 0.05
Women, %	37.8	52.2	63.6	75.5	< 0.05
Education ≥ 13 years, %	21.3	21.3	20.7	22.1	< 0.05
Hypertension, %	74.1	72.3	72.8	72.8	0.57
Diabetes, %	16.6	17.4	16.3	16.7	0.72
Serum total cholesterol, mg/dL	205 ± 37	207 ± 36	208 ± 36	207 ± 36	0.07
Body mass index, kg/m^2^	23 ± 3	23 ± 3	23 ± 3	23 ± 3	0.37
History of stroke, %	5.6	4.8	6.4	5.6	0.16
Smoking habit, %	50.6	37.0	30.1	18.6	< 0.05
Exercise habits, %	35.8	42.9	44.7	48.6	< 0.05
Energy intake, kcal/d	1799 ± 439	1843 ± 398	1864 ± 384	1878 ± 378	< 0.05

Values are shown as mean values ± standard deviations for continuous variable or frequencies for categorical variables General linear model and chi-square tests were used to test differences in risk factors across quartiles for each dietary pattern

### Association between dietary patterns and dementia

ORs and associated 95% CIs were estimated for the presence of dementia and its subtypes according to the score quartiles for five dietary patterns. Higher DP1 scores were significantly associated with a lower OR for dementia (Table 3). Subjects in the highest quartile of DP1 scores had a significantly lower prevalence of all-cause dementia compared with those in the lowest quartile, after adjusting for age and sex (OR = 0.47; 95% CI: 0.33, 0.65; *p* < 0.001) and after multivariate adjustments (OR = 0.56; 95% CI: 0.39, 0.79; *p* = 0.01). For AD, the OR similarly decreased after age and sex adjustment (OR = 0.40; 95% CI: 0.28, 0.58; *p* < 0.001) and after multivariate adjustments (OR = 0.47; 95% CI: 0.32, 0.69; *p* < 0.001). No significant differences were found for vascular dementia (VaD).

**Table 3:** Odds ratios (95% confidence intervals) of the presence of dementia according to the quartiles of the dietary pattern 1 score

	Score for the dietary pattern 1				
	Quartile 1	Quartile 2	Quartile 3	Quartile 4	*p*-value
**All-cause dementia**					
Events/population at risk (n)	98/2235	98/2234	87/2235	76/2234	—
Age and sex adjusted OR (95%CI)	1.00 (reference)	0.82 (0.60, 1.11)	0.63 (0.46, 0.87)	0.47 (0.33, 0.65)	< 0.01
Multivariate adjusted OR (95%CI)^1^	1.00 (reference)	0.90 (0.65, 1.24)	0.72 (0.51, 1.00)	0.56 (0.39, 0.79)	0.01
**Alzheimer disease**					
Events/population at risk (n)	80/2235	84/2234	70/2235	58/2234	—
Age and sex adjusted OR (95%CI)	1.00 (reference)	0.85 (0.61, 1.18)	0.59 (0.42, 0.84)	0.40 (0.28, 0.58)	< 0.01
Multivariate adjusted OR (95%CI)^1^	1.00 (reference)	0.91 (0.65, 1.28)	0.66 (0.46, 0.95)	0.47 (0.32, 0.69)	< 0.01
**Vascular dementia**					
Events/population at risk (n)	22/2235	10/2234	14/2235	18/2234	—
Age and sex adjusted OR (95%CI)	1.00 (reference)	0.39 (0.18, 0.83)	0.55 (0.28, 1.10)	0.70 (0.36, 1.34)	0.08
Multivariate adjusted OR (95%CI)^1^	1.00 (reference)	0.46 (0.21, 1.04)	0.63 (0.30, 1.32)	1.02 (0.49, 2.09)	0.16

OR, odds ratio; CI, confidence interval; Binary logistic regression was used to estimate the age- and sex adjusted or multivariate adjusted ORs (95%CIs)^1^ Adjusted for age, sex, education, diabetes, hypertension, serum total cholesterol, history of stroke, body mass index, smoking habits, exercise habits, and energy intake

Site-specific ORs and 95% CIs for DP1 were calculated across the eight study sites. A negative trend between higher DP1 scores and the prevalence of all-cause dementia (Supplementary data 2) and AD (Supplementary data 3) was observed in most sites, although statistical significance was not achieved in the majority of them. A positive trend was found only in the Hirosaki site.

### Association between DP1 and WMLs

Multiple linear regression was conducted to explore associations between WML volumes and the score quartiles for DP1 in all participants (Table 4). Volumes of all WML regions (total, PWML, DWML, and IWML) significantly decreased across increasing DP1 score quartiles after adjusting for eTIV, age, sex, and study site (Model 1; all *p* < 0.05). These associations remained significant after further adjustment for potential confounders (Model 2; all *p* < 0.05).

**Table 4:** Relationship between the quartiles of the Japanese diet including protein and minerals score and WML volume at different brain regions

	Quartiles of the Japanese diet including protein and minerals score		
	*R*-square	β	*p*-value
WML volume total			
Model 1	0.27	− 0.03	< 0.01
Model 2	0.30	− 0.03	< 0.01
WML volume periventricular			
Model 1	0.27	− 0.03	< 0.01
Model 2	0.30	− 0.03	< 0.01
WML volume deepwhite			
Model 1	0.23	− 0.04	0.02
Model 2	0.26	− 0.04	0.02
WML volume infracortical			
Model 1	0.12	− 0.05	0.01
Model 2	0.15	− 0.05	0.01

Multiple linear regression was employed to examine associations between dietary patterns and WML, Model 1: adjusted for eTIV, age, sex and study site, Model 2: adjusted for eTIV, age, sex, study site, education, diabetes, hypertension, serum total cholesterol, history of stroke, body mass index, smoking habits, exercise habits, and energy intake

In the subgroup of individuals without dementia, higher DP1 scores were also significantly associated with lower volumes of total WMLs, PWMLs, DWMLs, and IWMLs (Table 5). These associations persisted after full adjustment for covariates, supporting the robustness of the findings and suggesting that the relationship between DP1 and brain structural integrity may be independent of the presence of dementia.

**Table 5:** Relationship between the quartiles of the Japanese diet including protein and minerals score and WML volume at different brain regions among non-demented individuals

	Quartiles of the Japanese diet including protein and minerals score		
	*R*-square	β	*p*-value
WML volume total			
Model 1	0.25	− 0.03	< 0.01
Model 2	0.28	− 0.03	< 0.01
WML volume periventricular			
Model 1	0.25	− 0.03	< 0.01
Model 2	0.28	− 0.03	< 0.01
WML volume deepwhite			
Model 1	0.21	− 0.03	0.04
Model 2	0.24	− 0.03	0.03
WML volume infracortical			
Model 1	0.11	− 0.04	0.02
Model 2	0.13	− 0.05	0.02

Multiple linear regression was employed to examine associations between dietary patterns and WML, Model 1: adjusted for eTIV, age, sex and study site, Model 2: adjusted for eTIV, age, sex, study site, education, diabetes, hypertension, serum total cholesterol, history of stroke, body mass index, smoking habits, exercise habits, and energy intake

Additionally, to provide a more detailed view of the data and help contextualize potential confounding factors, plots illustrating the relationships between log-transformed WML volume and age, exercise habits, and MMSE total score are shown in Fig. S1–S3. A positive association is observed between age and WML volume, whereas a negative one is observed with MMSE scores.

## Discussion

We identified a dietary pattern associated with a lower prevalence of dementia and smaller volumes of WML in older Japanese people. This dietary pattern was characterized by high intakes of green and other vegetables, algae, potatoes, fish, fruits and fruit juices, egg, meat, pickles, soybeans and soybean products, milk and dairy products, and low intake of alcoholic drinks, sugar and confectioneries, roughly corresponding to a Japanese diet including protein and minerals. These findings highlight a dietary pattern that shows statistical associations with both dementia status and brain structural abnormalities in aging, contributing to the understanding of how diet may relate to cognitive and brain health in later life.

DP1 includes dietary components beneficial and potentially harmful to brain health. Vegetables, algae, soybeans, potatoes, and fruits contain dietary fiber, beneficial to brain health via gut-brain interactions involving microbiota [19]. Several studies have shown that soluble high-fiber foods induce neuroinflammation and dementia risk [20–22], consistent with our findings. Pickled foods may also positively influence gut microbiota and dementia risk [23]. Fish, seaweed, vegetables, and fruits provide beneficial nutrients, including n-3 polyunsaturated fatty acids, docosahexaenoic acid (DHA), eicosapentaenoic acid (EPA), folic acid, vitamin E, and antioxidants such as polyphenols and carotenoids. Eggs, meat, and dairy products have been reported to support cognitive function [24, 25], possibly due to their cholesterol content, essential for synapse formation and neuronal signaling [26]. Additionally, lutein, choline, zeaxanthin, and protein (abundant in eggs) effectively protect nerves, thereby preventing dementia [27]. Conversely, excessive alcohol intake is associated with dementia due to neurotoxic effects, cardiovascular complications, and thiamine deficiency [28]. High sugar consumption rapidly elevates blood glucose and insulin, potentially impairing brain function [29]. Given that excessive intake of cholesterol, pickles and fruit juices may be associated with a high risk of diabetes and cardiovascular disease, further research is needed to determine the optimal intake.

Several studies have reported that meat, fish, potatoes, vegetables, legumes, fruits, dairy products, and eggs, containing B vitamins, vitamin E, carotenoids, flavonoids, and long-chain omega-3 PUFAS, are associated with greater brain volume and lower WML volumes [30, 31]. These are consistent with our results. Conversely, inadequate intake of B vitamins (folate, B12, B6) increases the risk of hyperhomocysteinemia, contributing to white matter damage [32]. In addition, low serum levels of vitamin C (abundant in fruits, vegetables, and potatoes) and vitamin E (abundant in green vegetables) are associated with more severe DWML volumes [33]. In contrast, higher dietary intake of flavonoids (existing in fruits and legumes) was associated with lower WML volumes [34]. Fish is an excellent source of omega-3 fatty acids, particularly eicosapentaenoic acid (EPA) and docosahexaenoic acid (DHA), which are essential for neuronal integrity and may help reduce WML burden [35]. Meat, eggs, soy, and dairy provide quality protein for neuronal maintenance and repair [36, 37]. In contrast, excessive sugar and alcohol intake may trigger brain inflammation, oxidative stress, and metabolic disorders, leading to WMLs [38, 39].

WML volumes were quantified using a ConvNet model previously developed, which enables high-resolution detection of WMLs and thus enhances the validity of the study findings that a Japanese dietary pattern including minerals and proteins was associated with lower total WML volume.

WMLs were further categorized into PWML, DWML, and IWML regions based on their distinct etiological and clinical relevance. PWMLs are frequently linked to cerebrovascular dysfunction [40]. Thus, the inverse relationship between DP1 scores and PWML volumes may suggest that a healthier diet is related to reduced cerebrovascular burden. Additionally, DWMLs may be connected with metabolic syndrome, such as obesity [41]. Thus, dietary management might mitigate these metabolic risks and play a role in DWML development. Furthermore, IWMLs have been implicated in cognitive decline [42], and our findings suggest that this dietary pattern may be relevant to early neurodegenerative processes.

Importantly, a sensitivity analysis excluding individuals with dementia was conducted to reduce the potential for reverse causation due to prodromal dietary changes. The persistence of inverse associations between DP1 scores and WML volumes in this non-demented subgroup supports the hypothesis that diet is associated with brain health independent of dementia. Although the cross-sectional design limits causal inference, this sensitivity analysis enhances the biological plausibility of the observed associations. Since WMLs are considered early markers of cerebrovascular and neurodegenerative processes, these results suggest that favorable dietary patterns may be associated with better brain health even before the onset of clinical dementia. The consistent associations observed in the non-demented subgroup support the potential role of diet in cognitive and neurological health and help mitigate concerns regarding reverse causation in cross-sectional research.

Although the association between DP1 scores and log-transformed WML volume was relatively small (e.g., β for total WML volume = − 0.03), even minor variations in WML burden may carry clinical implications. Prior evidence has shown that the presence of WMLs is significantly associated with highly elevated risks of stroke, dementia, and all-cause mortality [43]. These associations underscore the clinical relevance of even mild WMLs and support their role as early indicators of future neurological decline.

Moreover, previous studies have consistently demonstrated that relatively low WML burden is associated with subtle but meaningful deterioration in multiple cognitive domains, including memory, processing speed, attention, and executive function [44]. These findings suggest that WML-related cognitive changes may begin early and gradually progress over time.

WMLs are also recognized as core neuroimaging markers of cerebral small vessel disease (CSVD), a major contributor to vascular cognitive impairment and stroke. Early-stage WMLs may reflect incipient microvascular damage that is potentially reversible, emphasizing their importance in early detection and preventive strategies [45].

From a public health perspective, although the individual-level effect may appear modest, the high prevalence of WMLs among older adults implies that even small associations could lead to a significant cumulative burden at the population level. Therefore, even mild WMLs may warrant consideration in clinical practice.

This study addresses several important gaps by focusing on a previously underrepresented population, applying high-resolution WMLs, and evaluating these associations within a specific cultural context.

This study represents the first standardized nationwide investigation in Japan to examine the associations between dietary patterns, dementia prevalence, and brain structural markers such as WMLs. Using a harmonized FFQ and uniform protocols, we analyzed data from 8,938 older adults recruited from eight study sites across Japan, ranging from Tohoku in the north to Kyushu in the south, encompassing diverse climatic and dietary environments.

Building on this nationwide framework, a site-specific analysis was conducted to evaluate the consistency and generalizability of the association between DP1 and dementia across Japan. Although an overall inverse association between DP1 scores and the prevalence of all-cause dementia and AD was observed in most study sites, statistical significance was not consistently achieved. One exception was Hirosaki, where a positive trend was likely due to the extremely small number of dementia cases.

The identification of a consistent dietary pattern (DP1) across regions and the application of subgroup analyses support the robustness and generalizability of this pattern across distinct regional contexts, distinguishing this study from previous investigations that were often limited in scale and scope.

Although the analytical methods used in this study, including PCA and deep learning-based WML quantification, have been applied in population-based studies, few studies have utilized these approaches in large-scale, multi-regional Japanese samples. This study enables the assessment of dietary patterns across diverse regions, improving the stability and internal consistency of the findings in Japan. The cultural specificity of dietary practices in Japan highlights the importance of conducting such population-specific investigations, rather than relying solely on evidence from different cultural and dietary contexts. These results contribute to the growing body of cross-cultural nutritional neuroepidemiology.

Another strength of the study is the introduction of a novel element by incorporating MRI-based neuroimaging markers through the high-resolution quantification of WMLs. While previous studies have linked specific foods or nutrients to brain structure, the interaction between overall dietary patterns and WMLs remains unclear, particularly within the context of Japanese dietary culture. To our knowledge, this is the first study in Japan to apply this imaging method in dietary pattern research. This approach, based on a validated ConvNet model, provides an objective and sensitive indicator of brain aging and allows for the assessment of associations between dietary behaviors and WMLs in older adults.

However, several limitations should be noted. First, a major limitation of this study is its cross-sectional design, which precludes conclusions about causal relationships between dietary patterns and WMLs or dementia. Although we conducted a sensitivity analysis excluding individuals with dementia in the analysis of dietary patterns and WMLs to reduce the risk of reverse causation, it remains possible that early or subclinical cognitive changes may have influenced dietary behaviors. This concern is particularly relevant in aging populations where lifestyle factors may be modified in response to subtle health changes. Therefore, the observed associations should be interpreted with care, and future longitudinal studies are warranted to investigate how adherence to this dietary pattern influences the development and progression of dementia and WML over time.

Second, dietary intake assessed by FFQ may introduce recall bias, although caregiver assistance was provided for participants with cognitive impairment. Last but not least, the study population consisted solely of older Japanese adults, which may limit the generalizability of the findings to populations with different dietary habits, genetic backgrounds, or age ranges.

## Conclusion

In this study, we found that a Japanese dietary pattern including protein and minerals was significantly associated with a lower prevalence of dementia and smaller WML volumes in older Japanese adults. Drawing on a standardized nationwide design and high-resolution WML quantification, this study contributes to the growing body of literature connecting diet to brain health. While these findings point to a correlation between culturally specific dietary patterns and brain aging, longitudinal and interventional studies are needed to clarify the direction and underlying mechanisms.

## Supplementary Information

Below is the link to the electronic supplementary material.

Supplementary data 1 (DOCX 16.9 kb)

Supplementary data 2 (DOCX 60.5 kb)

Supplementary data 3 (DOCX 60.7 kb)

Supplementary Fig. S1 Association between age and WML volume WML volume was log-transformed. A positive association is observed between age and WML volume. (PNG 493 kb)

High resolution image (TIF 30.8 mb)

Supplementary Fig. S2 Comparison of WML volume between individuals with and without exercise habits WML volume was log-transformed. Exercise habits were defined as engaging in physical activity (including walking) for at least 30 minutes per session, at least twice per week, and sustained for more than one year. (PNG 69.8 kb)

High resolution image (TIF 136 kb)

Supplementary Fig. S3 Association between MMSE total scores and WML volume WML volume was log-transformed. A negative association is observed between MMSE total scores and WML volume. (PNG 386 kb)

High resolution image (TIF 30.8 mb)

## Data Availability

The datasets used in the present study are not publicly available, because they contain confidential clinical data on the study participants. However, the data are available on reasonable request and with the permission of the Principal Investigator of this study, Toshiharu Ninomiya (ninomiya.toshiharu.734@m.kyushu-u.ac.jp).
