# Supplementary material for: From the plate to the brain: associations between dietary patterns and reduced dementia prevalence and white matter lesions in older Japanese adults
Source: GeroScience. 2025 Jul 29;48(2):2743–56. doi: 10.1007/s11357-025-01791-7 (PMC12972405; doi:10.1007/s11357-025-01791-7)
Supplement: Supplementary file 1 — (DOCX 16.9 kb) [file 11357_2025_1791_MOESM1_ESM.docx]

| **Supplementary data 1.** Food groups and food items included in the analysis of the JPSC-AD | |
| --- | --- |
| Food group | Definition and content |
| Green vegetables | Western pumpkin, canned tomato juice with salt, canned tomato juice without salt, canned carrot juice, green and yellow vegetables |
| Other vegetables | Fresh aloe vera leaf, dried shredded daikon radish, fresh garlic stems, pale colored vegetables |
| Algae | Roasted seaweed, seasoned seaweed, steamed and dried arame, shaved kombu seaweed, Tokoroten - Jelly-like Japanese dish, Tsukudani - preserved seaweed, fresh Mekabu wakame, seaweed |
| Potatoes | Jerusalem artichoke, konjac, sweet potato, steamed and dried sweet potato, raw taro root, dried glass noodles, tubers |
| Fish | Anchovy Tsukudani, dried anchovy, fresh dried sardine, semi-dried baby sardines, canned seasoned sardines, bonito flakes, salmon roe, canned boiled salmon, canned mackerel in miso, canned seasoned mackerel, canned seasoned tuna flakes, dried sakura shrimp, dried squid strips, fresh krill, white fish, blue fish, red fish, shellfish, processed seafood products |
| Rice | Pressed barley, roasted barley, brown rice, half-milled rice, seven-tenths milled rice, white rice, rice polished with bran, sprouted brown rice, rice koji, mochi, rice bran, cereal grains, fortified rice |
| Noodles and other cereals | boiled udon, boiled somen and hiyamugi, boiled chinese-style noodles, instant fried chinese-style noodles, instant cup noodles, boiled macaroni & spaghetti, general noodle types, whole grain buckwheat flour, boiled soba noodles, barley oatmeal, okonomiyaki premix flour, cornflakes, fruit granola, soy granola |
| Bread | Bread |
| Alcoholic drinks | Regular sake, light beer, red wine, Shaoxing wine, single distillation shochu, whiskey, vodka |
| Sugar and confectioneries | Black sugar, Wasanbon Sugar, granulated sugar, brown sugar, granulated sugar, medium grain sugar, fructose glucose syrup, black syrup, honey, maple syrup, kuzumanju - japanese sweet, chinese-style steamed bun with meat filling, candy drops, soy sauce rice crackers, melon bread, shortcake without fruit, orange jelly, soda crackers, potato chips, milk chocolate, general confectionery |
| Fruits and fruit juices | Avocado, high sugar apricot jam, high sugar strawberry jam, low sugar strawberry jam, salt-pickled plums, seasoned pickled plums, salted umeboshi - dried plums, seasoned Umeboshi - dried plums, dried Persimmons, concentrated orange juice, 30% orange juice drink, high sugar orange marmalade, fresh yuzu peel, coconut milk, dried prunes, dried grapes/raisins, grape jam, blueberry jam, dried blueberries, peeled fresh apple, apple jam, fruits |
| Fats and oils | Amani oil, perilla oil, olive oil, sesame oil, rice bran oil, high oleic safflower oil, blended oil, canola oil, grape seed oil, palm oil, salted butter, soft type margarine for home use |
| Egg | Raw whole chicken egg, raw egg yolk, raw egg white |
| Meat | Raw horse meat, pork gelatin, raw fatty pork back fat, raw chicken liver, beef, pork, chicken, processed meat products |
| Pickles | Napa cabbage kimchi, sweet vinegared scallions, old pickles, light pickles |
| Soybeans and soybean products | Dried whole azuki beans, boiled whole azuki beans, chunky azuki bean paste, pigeon peas, boiled soybeans, roasted yellow soybeans, roasted black soybeans, steamed yellow soybeans, soybean flour, soybeans in grape shape, stringy natto, soy milk, processed soy milk, soy milk beverage/malt coffee, yuba - soy milk skin, kinzanji miso, boiled lentils |
| Milk and dairy products | Regular cow's milk, processed milk (high concentration), low-fat processed milk, skimmed milk, milk beverage with coffee, skimmed milk powder, prepared milk powder for infants, sweetened condensed milk, whipped cream (milk fat), coffee whitener (milk and vegetable fat), coffee whitener (vegetable fat), powdered coffee whitener (milk fat), full-fat unsweetened yogurt, low-fat unsweetened yogurt, skimmed sweetened yogurt, lactic acid bacteria beverage (dairy product), regular fat ice cream, milk and dairy products sherbet, cheese |
| Miso | Sweet rice miso, light-colored spicy rice miso, red-colored spicy rice miso, barley miso, bean miso, vinegared miso |
