# Supplementary figures and images for: From the plate to the brain: associations between dietary patterns and reduced dementia prevalence and white matter lesions in older Japanese adults
Source: GeroScience. 2025 Jul 29;48(2):2743–56. doi: 10.1007/s11357-025-01791-7 (PMC12972405; doi:10.1007/s11357-025-01791-7)

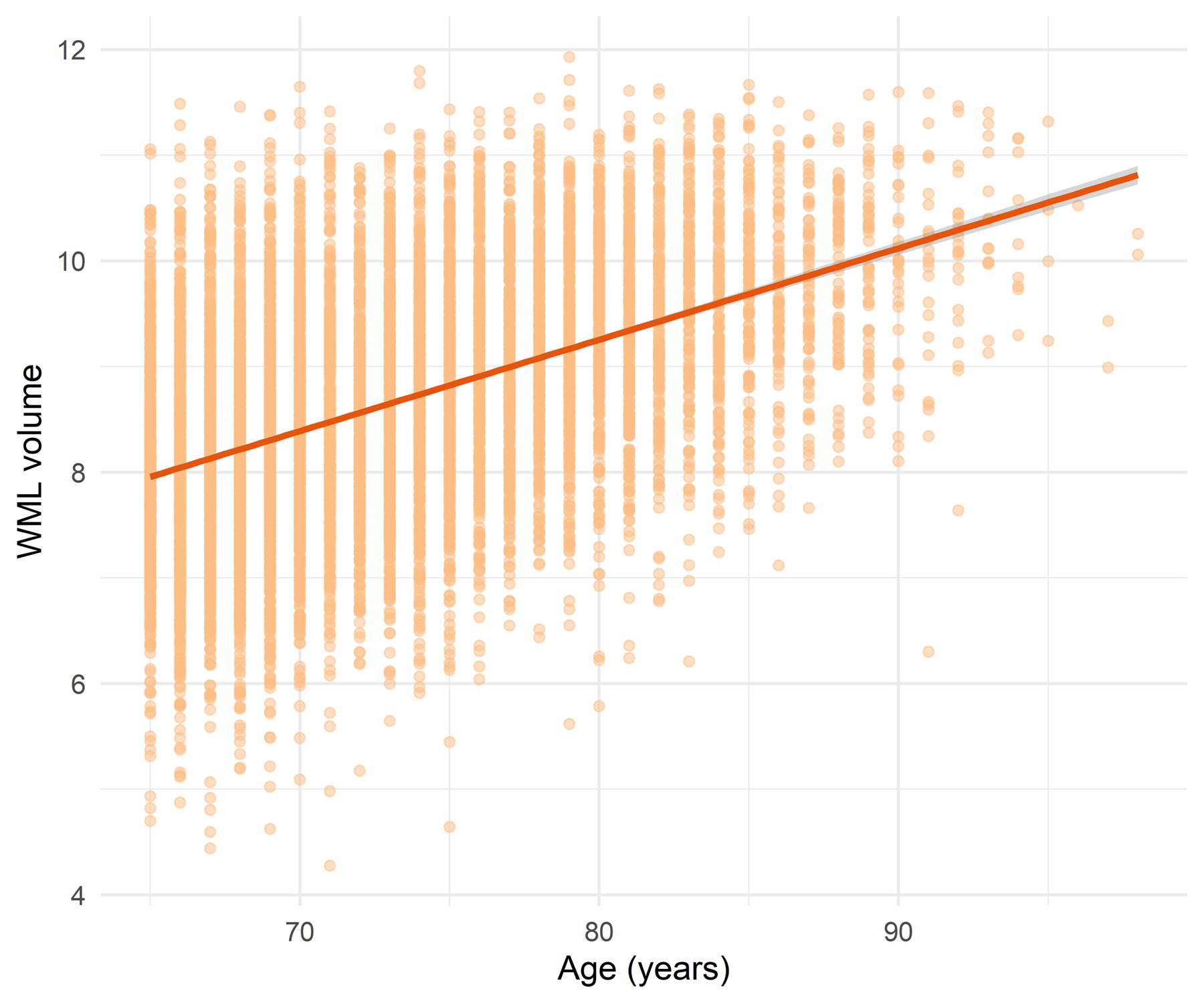

Supplement: Supplementary file 4 — Association between age and WML volume WML volume was log-transformed. A positive association is observed between age and WML volume. (PNG 493 kb) [file 11357_2025_1791_Fig3_ESM.png]

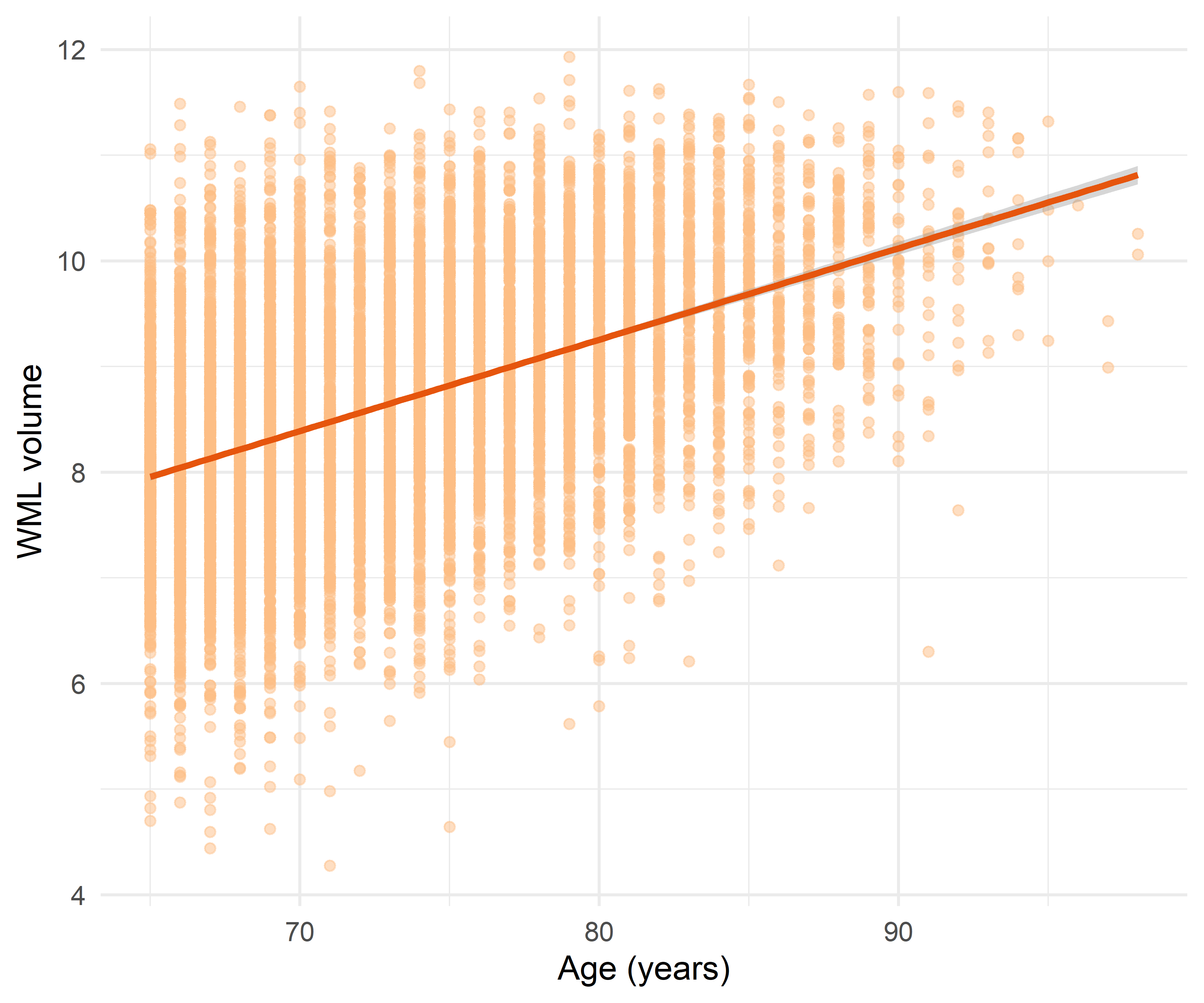

Supplement: Supplementary file 5 — High resolution image (TIF 30.8 mb) [file 11357_2025_1791_MOESM4_ESM.tiff]

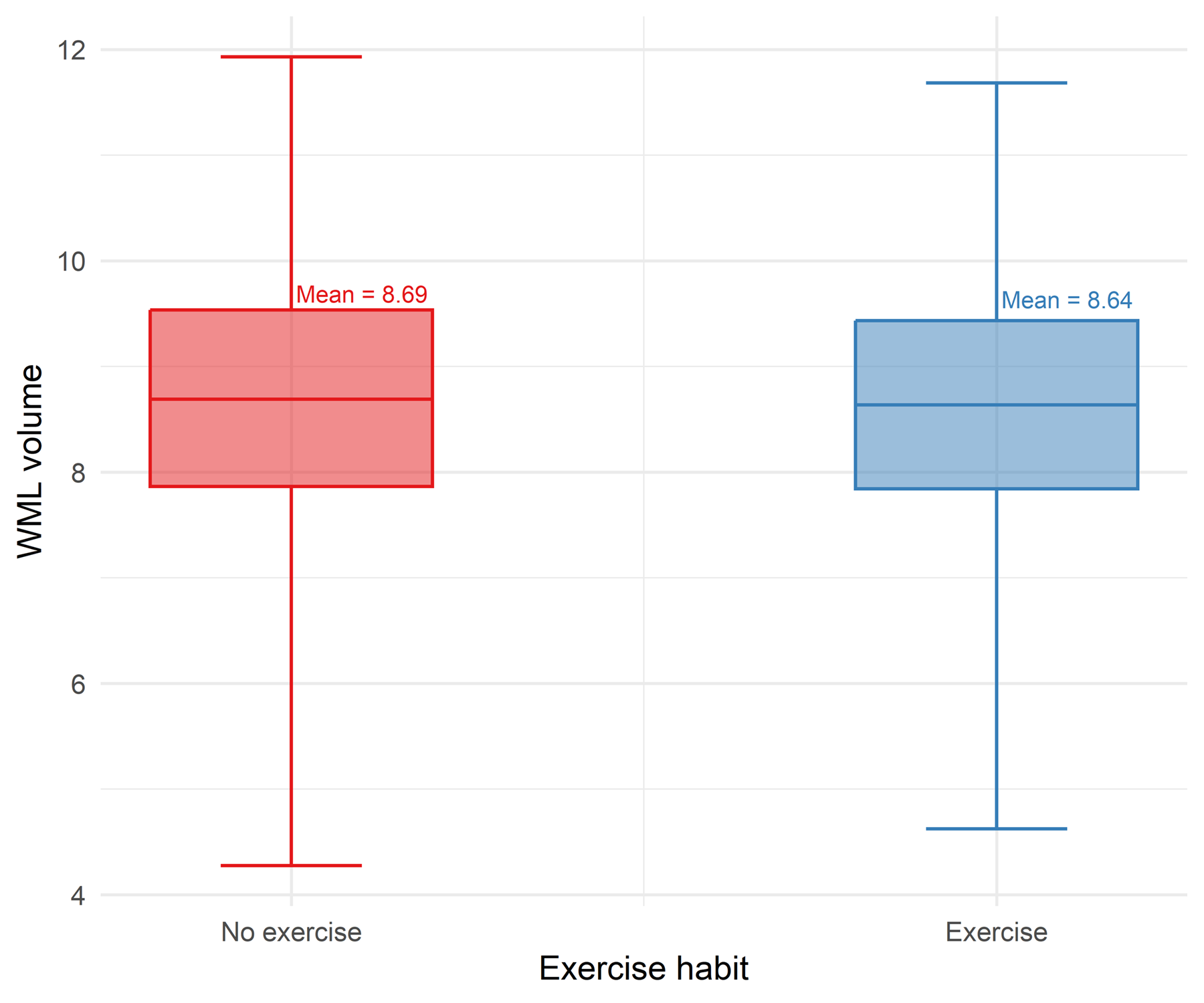

Supplement: Supplementary file 6 — Comparison of WML volume between individuals with and without exercise habits WML volume was log-transformed. Exercise habits were defined as engaging in physical activity (including walking) for at least 30 minutes per session, at least twice per week, and sustained for more than one year. (PNG 69.8 kb) [file 11357_2025_1791_Fig4_ESM.png]

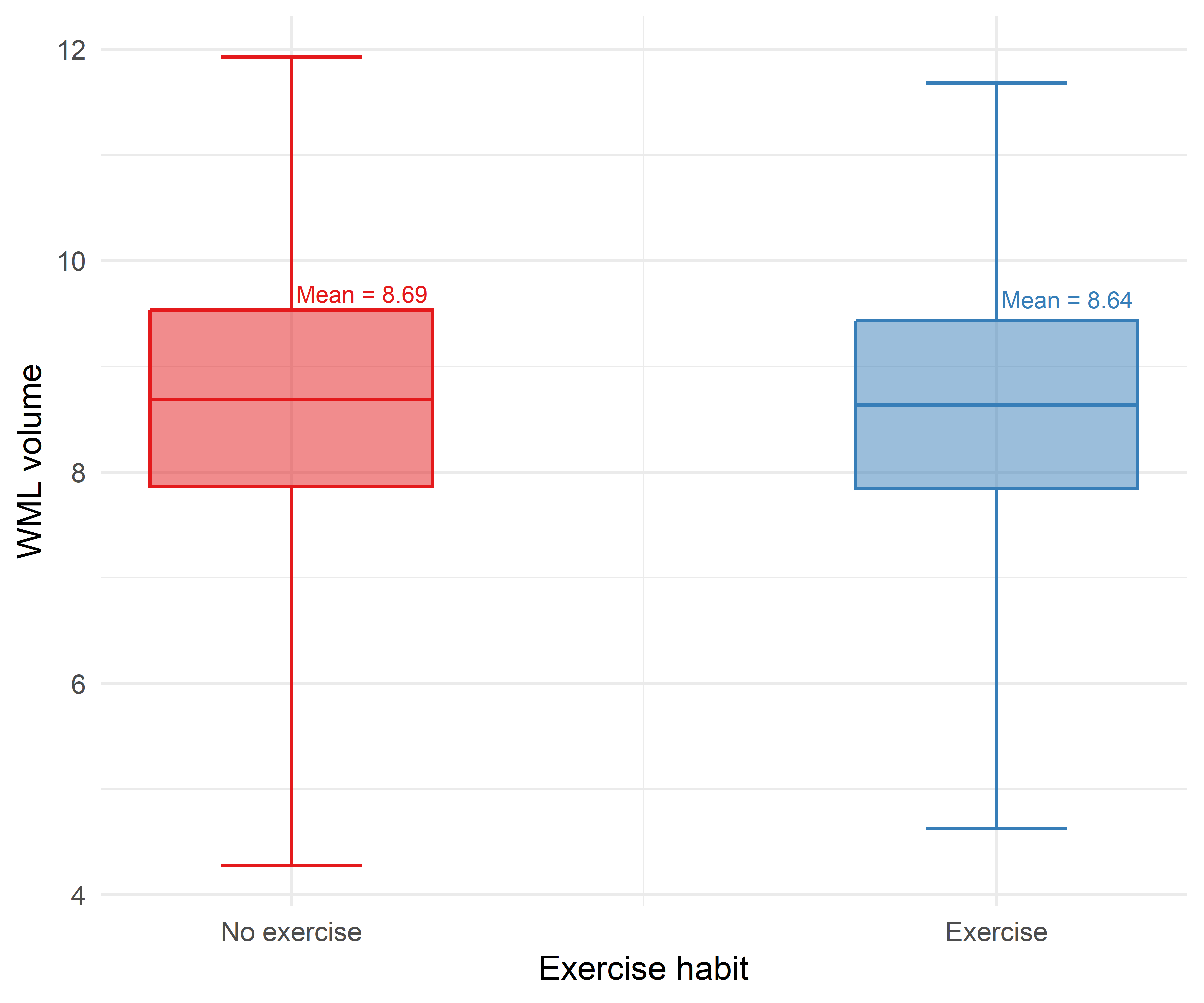

Supplement: Supplementary file 7 — High resolution image (TIF 136 kb) [file 11357_2025_1791_MOESM5_ESM.tiff]

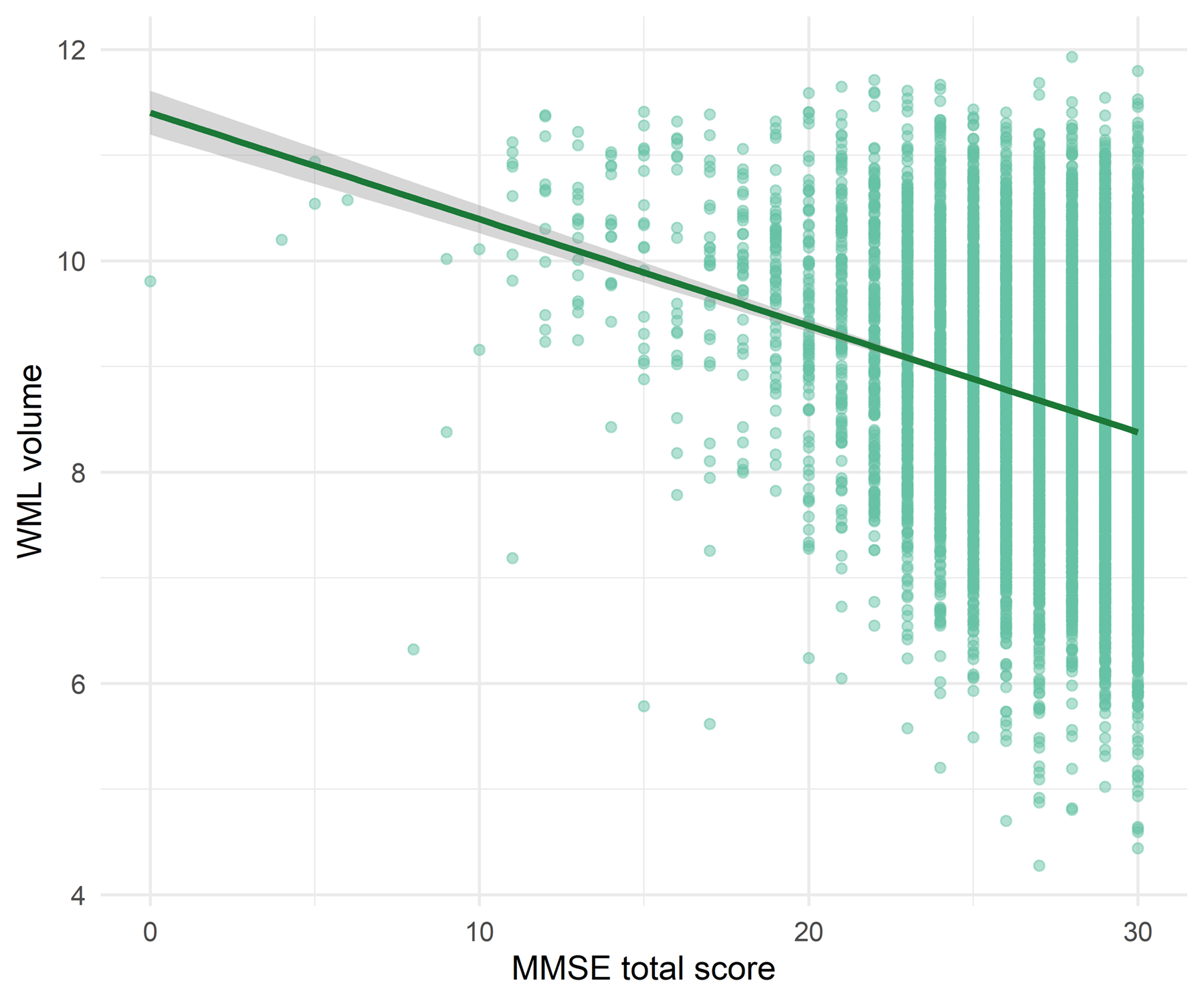

Supplement: Supplementary file 8 — Association between MMSE total scores and WML volume WML volume was log-transformed. A negative association is observed between MMSE total scores and WML volume. (PNG 386 kb) [file 11357_2025_1791_Fig5_ESM.png]

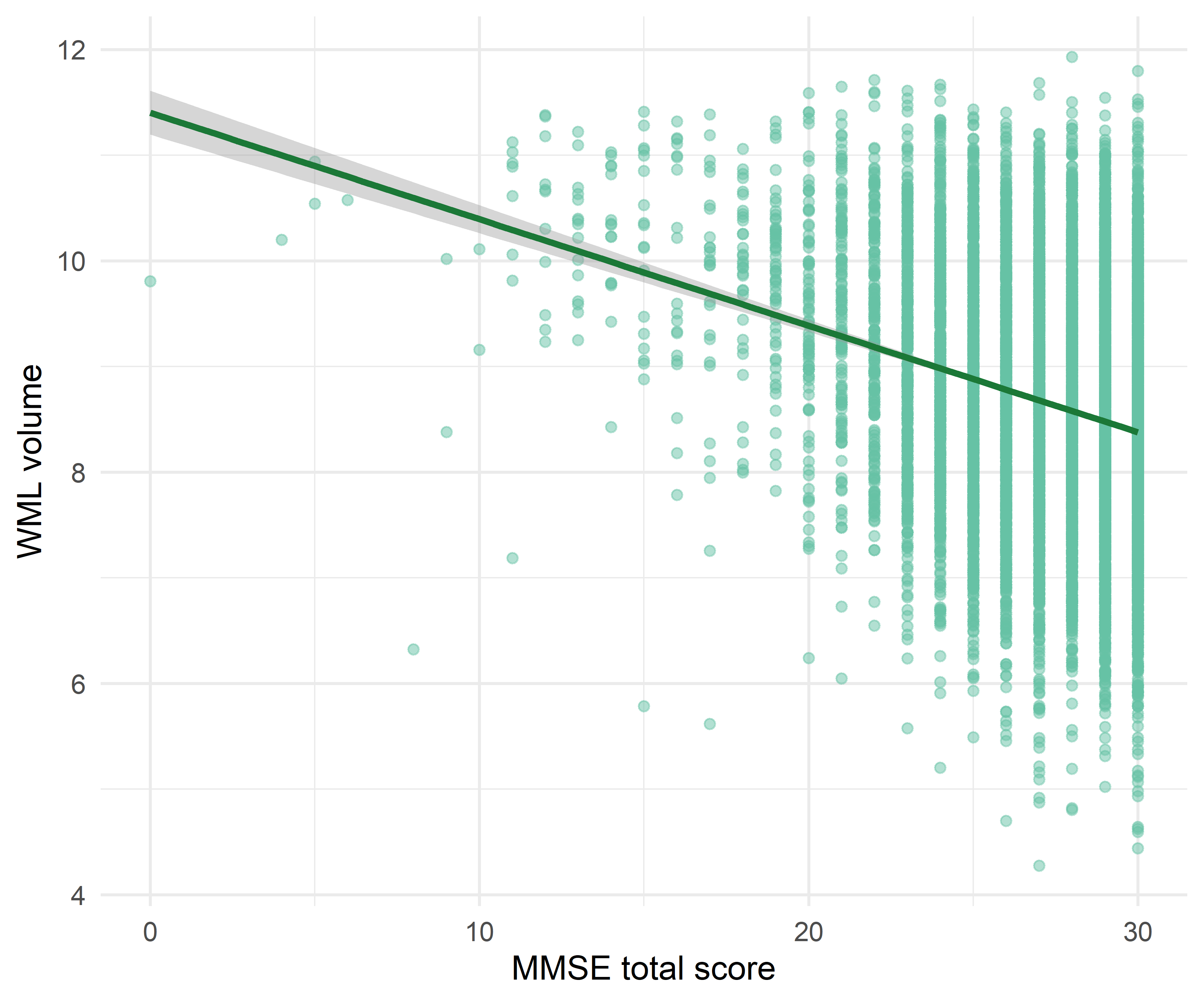

Supplement: Supplementary file 9 — High resolution image (TIF 30.8 mb) [file 11357_2025_1791_MOESM6_ESM.tiff]
