# Supplementary material for: From the plate to the brain: associations between dietary patterns and reduced dementia prevalence and white matter lesions in older Japanese adults
Source: GeroScience. 2025 Jul 29;48(2):2743–56. doi: 10.1007/s11357-025-01791-7 (PMC12972405; doi:10.1007/s11357-025-01791-7)
Supplement: Supplementary file 3 [file 11357_2025_1791_MOESM2_ESM.docx]

Supplementary data 2. Odds ratios (95% confidence intervals) of the prese
cause dementia across the eight sites according to the dietary pattern 1 score

	Score for the dietary pattern 1			
	Quartile 1	Quartile 2	Quartile 3	Qua
**Site1** (Ehime, n=687)				
Events/population at risk (n)	23/164	20/172	14/177	16
Age and sex adjusted OR	1.00 (reference)	0.72 (0.34, 1.45)	0.40 (0.19, 0.86)	0.44<br>0.9
Multivariate adjusted OR[1]	1.00 (reference)	0.89 (0.42, 1.91)	0.42 (0.19, 0.97)	0.61 (0
**Site2** (Hirosaki, n=2150)				
Events/population at risk (n)	1/570	3/544	2/560	4/
Age and sex adjusted OR	1.00 (reference)	3.19 (0.33,30.99)	2.08 (0.18,23.65)	4.84<br>46
Multivariate adjusted OR[1]	1.00 (reference)	3.09 (0.30,31.52)	1.73 (0.14,21.14)	3.11<br>32
**Site3** (Kyushu, n=1401)				
Events/population at risk (n)	25/358	25/375	23/351	24
Age and sex adjusted OR	1.00 (reference)	0.61 (0.40, 1.40)	0.57 (0.30, 1.09)	0.61<br>1.
Multivariate adjusted OR[1]	1.00 (reference)	0.84 (0.44, 1.61)	0.69 (0.35, 1.37)	0.82<br>1.
**Site4** (Iwate, n=906)				
Events/population at risk (n)	11/125	8/182	16/246	10
Age and sex adjusted OR	1.00 (reference)	0.36 (0.13, 0.99)	0.52 (0.22, 1.24)	0.17<br>0.
Multivariate adjusted OR[1]	1.00 (reference)	0.46 (0.16, 1.34)	0.61 (0.24, 1.58)	0.21<br>0.
**Site5** (Kanazawa, n=813)				
Events/population at risk (n)	11/207	8/203	9/190	5/
Age and sex adjusted OR	1.00 (reference)	0.58 (0.22, 1.56)	0.65 (0.25, 1.70)	0.24<br>0.
Multivariate adjusted OR[1]	1.00 (reference)	0.47 (0.17, 1.33)	0.52 (0.18, 1.49)	0.22<br>0.
**Site6** (Keio, n=992 subjects)				
