# Supplementary material for: From the plate to the brain: associations between dietary patterns and reduced dementia prevalence and white matter lesions in older Japanese adults
Source: GeroScience. 2025 Jul 29;48(2):2743–56. doi: 10.1007/s11357-025-01791-7 (PMC12972405; doi:10.1007/s11357-025-01791-7)
Supplement: Supplementary file 4 [file 11357_2025_1791_MOESM3_ESM.docx]

Supplementary data 3. Odds ratios (95% confidence intervals) of the preser[illegible] Alzheimer disease across the eight sites according to the dietary pattern 1 sco[illegible]

	Score for the dietary pattern 1			
	Quartile 1	Quartile 2	Quartile 3	Qua[illegible]
**Site1** (Ehime, n=687)				
Events/population at risk (n)	14/164	16/172	10/177	10[illegible]
Age and sex adjusted OR	1.00 (reference)	1.03 (0.46, 2.32)	0.51 (0.21, 1.26)	0.48[illegible] 1.[illegible]
Multivariate adjusted OR[1]	1.00 (reference)	1.32 (0.55, 3.17)	0.59 (0.22, 1.54)	0.65 (0[illegible]
**Site2** (Hirosaki, n=2150)				
Events/population at risk (n)	1/570	3/544	2/560	2/[illegible]
Age and sex adjusted OR	1.00 (reference)	3.09 (0.32, 30.04)	1.94 (0.17, 22.21)	2.18[illegible] 26.[illegible]
Multivariate adjusted OR[1]	1.00 (reference)	2.69 (0.27, 27.27)	1.79 (0.15, 21.79)	1.62[illegible] 20.[illegible]
**Site3** (Kyushu, n=1401)				
Events/population at risk (n)	23/358	22/375	17/351	19[illegible]
Age and sex adjusted OR	1.00 (reference)	0.69 (0.36, 1.33)	0.41 (0.20, 0.84)	0.46[illegible] 0.[illegible]
Multivariate adjusted OR[1]	1.00 (reference)	0.72 (0.37, 1.41)	0.44 (0.21, 0.93)	0.51[illegible] 1.[illegible]
**Site4** (Iwate, n=906)				
Events/population at risk (n)	10/125	7/182	14/246	10[illegible]
Age and sex adjusted OR	1.00 (reference)	0.35 (0.12, 1.00)	0.49 (0.20, 1.23)	0.19[illegible] 0.[illegible]
Multivariate adjusted OR[1]	1.00 (reference)	0.46 (0.15, 1.44)	0.60 (0.22, 1.66)	0.24[illegible] 0.[illegible]
**Site5** (Kanazawa, n=813)				
Events/population at risk (n)	9/207	6/203	8/190	5/[illegible]
Age and sex adjusted OR	1.00 (reference)	0.53 (0.17, 1.65)	0.72 (0.25, 2.05)	0.28[illegible] 0.[illegible]
Multivariate adjusted OR[1]	1.00 (reference)	0.43 (0.13, 1.41)	0.63 (0.20, 2.00)	0.26[illegible] 1.[illegible]
**Site6** (Keio, n=992)				
Events/population at risk (n)	8/344	8/273	4/220	3/[illegible]
